# Quantification of Scheduling Impact on Safety and Efficacy Outcomes of Brain Metastasis Radio- and Immuno-Therapies: A Systematic Review and Meta-Analysis

**DOI:** 10.3389/fonc.2020.01609

**Published:** 2020-09-02

**Authors:** Veronika Voronova, Svetlana Lebedeva, Marina Sekacheva, Gabriel Helmlinger, Kirill Peskov

**Affiliations:** ^1^M&S Decisions LLC, Moscow, Russia; ^2^Institute of Pharmacy, I.M. Sechenov First Moscow State Medical University, Moscow, Russia; ^3^Computational Oncology Group, I.M. Sechenov First Moscow State Medical University, Moscow, Russia; ^4^Clinical Pharmacology and Toxicology, Obsidian Therapeutics, Cambridge, MA, United States

**Keywords:** radiotherapy, immunotherapy, meta-analysis, immune checkpoints, treatment scheduling, brain metastases

## Abstract

**Objectives:** The goal of this quantitative research was to evaluate the impact of various factors (e.g., scheduling or radiotherapy (RT) type) on outcomes for RT vs. RT in combination with immune checkpoint inhibitors (ICI), in the treatment of brain metastases, via a meta-analysis.

**Methods:** Clinical studies with at least one ICI+RT treatment combination arm with brain metastasis patients were identified via a systematic literature search. Data on 1-year overall survival (OS), 1-year local control (LC) and radionecrosis rate (RNR) were extracted; for combination studies which included an RT monotherapy arm, odds ratios (OR) for the aforementioned endpoints were additionally calculated and analyzed. Mixed-effects meta-analysis models were tested to evaluate impact on outcome, for different factors such as combination treatment scheduling and the type of ICI or RT used.

**Results:** 40 studies representing a total of 4,359 patients were identified. Higher 1-year OS was observed in ICI and RT combination vs. RT alone, with corresponding incidence rates of 59% [95% CI: 54-63%] vs. 32% [95% CI: 25-39%] (*P* < 0.001). Concurrent ICI and RT treatment was associated with significantly higher 1-year OS vs. sequential combinations: 68% [95% CI: 60-75%] vs. 54% [95% CI: 47-61%]. No statistically significant differences were observed in 1-year LC and RNR, when comparing combinations vs. RT monotherapies, with 1-year LC rates of 68% [95% CI: 40-90%] vs. 72% [95% CI: 63-80%] (*P* = 0.73) and RNR rates of 6% [95% CI: 2-13%] vs. 9% [95% CI: 5-14%] (*P* = 0.37).

**Conclusions:** A comprehensive, study-level meta-analysis of brain metastasis disease treatments suggest that combinations of RT and ICI result in higher OS, yet comparable neurotoxicity profiles vs. RT alone, with a superiority of concurrent vs. sequential combination regimens. A similar meta-analysis using patient-level data from past trials, as well as future prospective randomized trials would help confirming these findings.

## Introduction

Data from numerous preclinical studies collected over the past 30 years have pointed to a pivotal role of the immune system in the anticancer efficacy of radiotherapy (RT). In landmark experiments by Helen Stone et al., the radiation dose required to achieve tumor control in 50% of mice was 1.7-fold higher in immuno-suppressed vs. immuno-competent animals (1). Subsequently, studies by Demaria et al. have shown potentiation of the RT abscopal effect (reduction of non-irradiated tumor lesions) in mice treated with RT and dendritic cell growth factor (2). More recent, preclinical experiments revealed factors which would limit the efficacy of RT-triggered antitumor immune response; it has been shown that immune checkpoints represent an important negative feedback downregulating T-lymphocytes function, thereby providing a biological rationale for combining RT with immune checkpoint inhibitors (ICI) (3, 4).

The clinical efficacy of RT and ICI combinations has been reviewed in a number of retrospective analyses and case reports, with a focus on the treatment of central nervous system (CNS) metastatic disease (5–7). The rationale for this was provided by clinical observations indicative of high lymphocyte infiltration into brain metastases, which pointed to the CNS as an “immune privileged” tissue with immunoregulatory microenvironment and motivated further prospective testing of RT and ICI combinations (8). In line with such observations, a high rate of intracranial response in patients treated with ICI combination has been demonstrated in a Phase II clinical study (CheckMate 204), reinforcing interest in using ICI for the management of brain metastases (9).

However, despite initial gains in treatment outcomes, numbers of responding patients still remain rather low, and further treatment optimization is warranted in order to derive incremental therapeutic benefits (10, 11). As can be concluded from preclinical experiments, numerous factors such as RT dose and fractionation regimen, the class of ICI, as well as the relative timing (scheduling) of ICI vs. RT therapy may all affect treatment outcomes (12). To understand and quantify the influence of such factors on preclinical efficacy of ICI-RT combinations, we used a mathematical mechanistic modeling approach to demonstrate superiority of concurrent vs. sequential ICI-RT administration (13). In the clinical setting, however, the interpretation of data across trials can be challenging due to variabilities in study designs, therapeutic options and patients enrolled. To address these challenges and qualify our earlier preclinical findings with clinical data, we here used a meta-regression modeling approach.

## Materials and Methods

### Literature Review and Data Collection

The current work was performed using the *Preferred Reporting Items for Systematic Review and Meta-analyses* (PRISMA) and *Meta-analysis of Observational Studies in Epidemiology* (MOOSE) guidelines (14, 15). PRISM and MOOSE checklists are available in Supplementary Tables 1, 2. A systematic literature search was conducted independently by two investigators (V.V. and K.P.) *via* an assessment of the PubMed database and publication materials from clinical oncology conferences (*American Society of Clinical Oncology* (ASCO), *European Society for Medical Oncology* (ESMO), *American Society for Radiation Oncology* (ASTRO) meetings). Original articles and abstracts reporting efficacy and safety outcomes of RT and ICI combinations published up to June 2019 were identified. The following keywords were used to conduct the search: (radiotherapy OR radiosurgery) AND (immunotherapy OR nivolumab OR pembrolizumab OR atezolizumab OR durvalumab OR ipilimumab OR tremelimumab OR lambrolizumab OR ticilimumab OR cemiplimab OR PD-1 OR PD-L1 OR CTLA-4) AND (brain AND metastases).

### Eligibility Criteria

The following eligibility criteria were used for study selection into the meta-analysis: conference abstracts and published or accepted manuscripts in English language reporting results of retrospective analyses and, if available, randomized clinical trials, enrolling patients with brain metastases of any origin treated with RT in combination with ICI; and including information on at least one of the considered efficacy or safety outcomes. Studies with <10 patients were excluded from the analysis. Also, if several combination arms with *N* < 10 were reported within one arm, information from these arms was pooled.

### Outcome Measures

Milestone overall survival (OS) and local control (LC) were selected as efficacy endpoints. A cut-off time of 1 year was set to yield a sufficient number of observations while maintaining a relatively small number of censored events (OS and LC were calculated from the start of ICI or RT). Local control was defined as the percentage of patients with radiographic decrease, or <20% increase in the size of irradiated lesions (16). Values of milestone OS and LC were digitized from Kaplan-Meier curves or extracted from manuscript texts or tables; information on censored events at cut-off times was extracted from the manuscripts. Radionecrosis rate (RNR) was selected as a safety outcome. Definitions of RNR differed across the collected studies and involved imaging and/or histological examinations as well as worsening of neurologic symptoms (17–19). For studies reporting RT alone arm as an active control, odds ratios (OR) for the outcomes were calculated, given the RT monotherapy arm as an active control.

### Data Considerations

Study characteristics such as median follow-up times, histological features of treated brain metastases, as well as treatment regimens—including type of RT and ICI treatments—and relative timings of RT vs. ICI therapies were all considered. Treatment regimens were classified as “RT alone” or “ICI-RT” (for combinations). “ICI-RT” treatment was further classified as “concurrent ICI-RT” if ICI treatment was administered within 4 weeks of the start or end of RT, otherwise it was classified as “sequential ICI-RT”; the threshold was selected based on the half-lives of ICI drugs (20, 21). If information on such timing was not reported or alternative cut-offs were used, the regimen was classified as “unknown ICI-RT.” ICI therapy was classified as “PD-(L)1” or “CTLA-4” targeted; if both ICI classes or their combination were considered, the therapy was defined as “unknown ICI-RT.” If only stereotactic RT regimens such as SRS or stereotactic radiotherapy (SRT) were used in the study, they were classified as “stereotactic”; if other conventional RT regimens such as whole-brain radiotherapy (WBRT), partial brain irradiation (PBI) were also considered, the RT regimen was classified as “WBRT-PBI.” Patient characteristics were collected, including age, histology, initial tumor volume, Karnofsky performance status (KPS) or ECOG performance status, previous chemotherapy (CT) or targeted therapy with BRAF inhibitors, combination of BRAF and MEK inhibitors, EGFR or ALK inhibitors or other agents (TT). If patient data were reported for each group separately, a weighted average for the entire study was calculated. Some important patient characteristics such as previous form of treatment and performance status were reported in heterogeneous formats or were not reported (majority of conference abstracts); this would then limit the corresponding data extraction and, subsequently, extensive covariate testing.

### Statistical Methods

The meta-analysis was performed in the R-based package *metaphor* (R version 3.5.1, metafor version 2.1-0) using the DerSimonian-Laird estimator (22). An arcsine square-root transformation was applied to incidence rates, whereas OR were log-transformed prior to the analysis for variance stabilizing (23). Based on low dropout rates during the first year of study (Supplementary Figure 1), confidence intervals for outcomes (CI) were estimated using the direct method as described in (24); the effect of censoring on the 1-year LC measure was not taken into account, since such data were not reported for some of the studies. The appropriateness of the following assumptions was evaluated during a covariate search stage, via testing of the censoring number as a continuous covariate (Supplementary Table 4). The Cochran Q test and the I-square (I^2^) test were used to assess heterogeneity across studies (22). A sensitivity analysis was also performed and included an assessment of publication bias for each outcome using funnel plots and the Egger test (22).

The meta-analysis was conducted in two stages: firstly, the difference between cohorts treated with RT alone vs. RT and ICI combinations was evaluated *via* an analysis of OR and incidence rates; secondly, the evaluation of additional factors (e.g., treatment regimen, RT, or ICI type) on the outcomes measures was investigated *via* mixed-effects meta-regression. A sequential (forward and backward) step-wise covariate search procedure was used, as described by Hutmacher and Kowalski (25). The final model was chosen based on multiple criteria, including the value of the Akaike information criterion with correction for small sample size (AICc), confidence intervals (CIs) of the regression coefficients, and different model diagnostic plots. Details of the meta-regression modeling and study level characteristics are all presented in Supplementary Tables 3, 4.

## Results

### Study Selection and Characteristics

**Figure 1:**
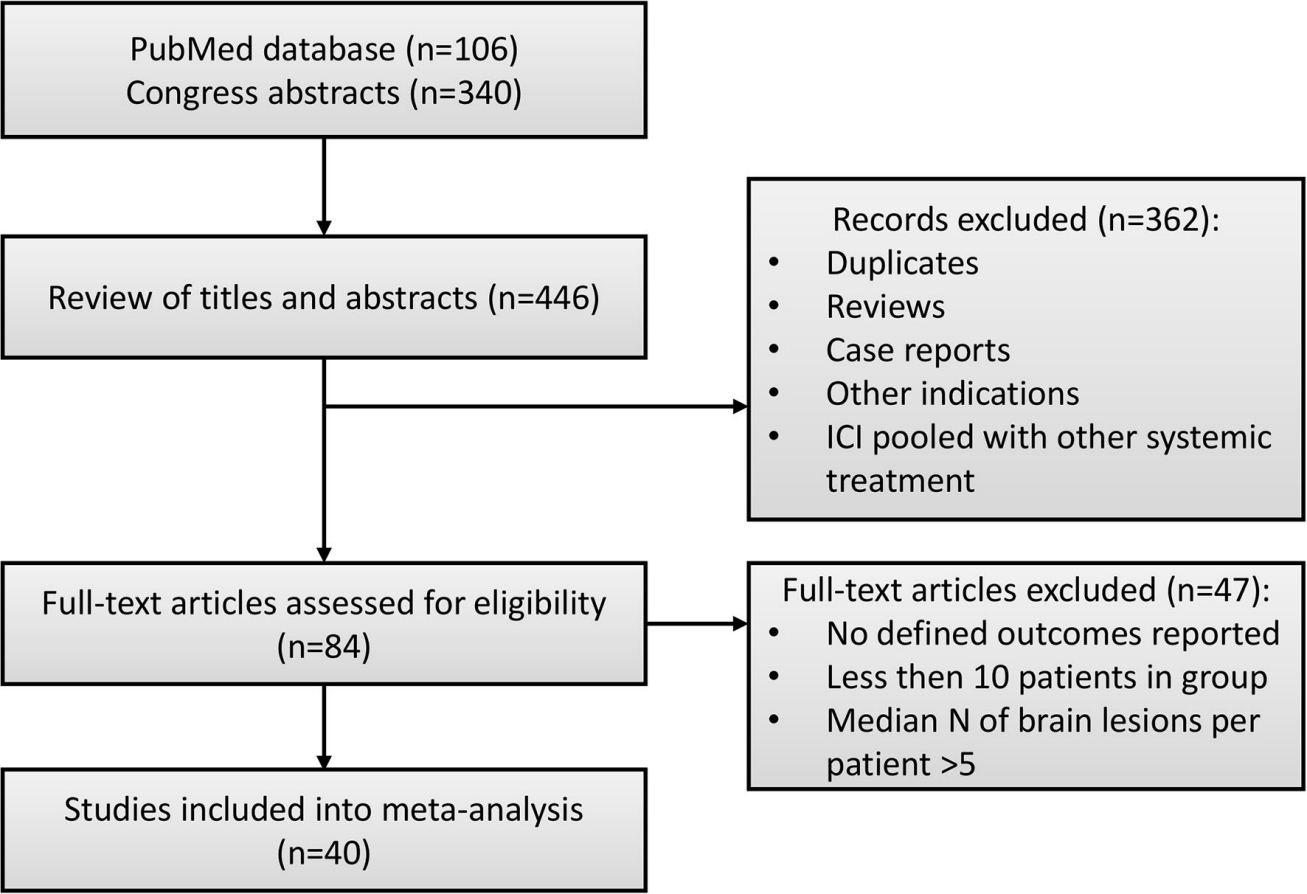
Flow diagram of study selection for quantitative meta-analysis.

Details of the study selection process are presented in Figure 1. In total, 446 studies were identified via a systematic literature search. Duplicates, reviews and case reports, and studies that focused on other indications (e.g., non-brain metastases) or pooled results of ICI and RT with other systemic treatments (e.g., BRAF/MEK, ALK inhibitors) were excluded from further analysis. In total, 84 full-text manuscripts and conference papers were evaluated. Since median OS or PFS were reported without CIs in the majority of studies, we based our analysis on milestone OS measures, thus only studies reporting such measures were included into the final analysis dataset. For similar reasons, objective response rate (ORR) data were not included into the final dataset, since this measure was reported in only 8 studies, moreover with different response criteria used across these, including response evaluation criteria in solid tumors (RECIST) (26), criteria proposed by the Response Assessment in Neuro-Oncology Brain Metastases (RANO-BM) working group (21, 27) and World Health Organization (WHO) (28), and immune-related response criteria (irRC) (26, 28–30). Information on distant intracranial control was not included either, since it was reported in 8 studies only and measured using different methods, making these data not suitable for data pooling. Finally, studies by Gerber et al. and Lopez-Martin et al. were excluded from the final dataset, since these studies were enrolling patients with high numbers of metastases, which significantly affected summary OS measures. In total, 40 studies with 4,359 patients, with reported 1-year OS, 1-year LC and RNR were included in this analysis (16–20, 26–60); most sources were retrospective analyses and only two publications reported results from prospective randomized clinical trials (29, 58).

A summary of the studies considered is detailed in Table 1; a summary of covariate distributions is shown in Supplementary Figure 1. The tumor types tested included brain metastases originating from melanoma (*N* = 33), NSCLC (*N* = 2), and pooled solid tumors (mainly melanoma and NSCLC, *N* = 5), which can be explained by the broad use of ICI drugs in the treatment of advanced melanoma and NSCLC combined with a high rate of brain metastases observed in these indications (62). Stereotactic RT regimens only were used in 29 studies; in the remaining 11 studies, conventional RT regimens were also considered. Anti CTLA-4 antibodies (Ab), anti PD-(L)1 Ab, or both classes of agents were tested in, respectively, 13, 13, and 14 studies. Information on treatment sequence was available in 14 studies and 20 arms, of which 10 arms were treated concurrently. Information on censoring was available in 25 studies; the median 1-year censoring rate was 10% (Supplementary Figure 2).

**Table 1:** Patient and study characteristics.

**References**	**N (*P*)**	**Median N (tL)**	**Median FU, months**	**Age**	**Histology**	**GTV median**	**KPS≥90, ECOG PS = 0**	**RT type (Median dose)**	**ICI target (Drug)**
Ahmed et al. (16)	26	2	9.4	54.5	MBM	0.22	62	SRS (21 Gy), FSRT (30 Gy)	PD-(L)1 (N)
Qian et al. (40)	55	4	15.5	62.5	MBM	0.11	NR	SRS (20 Gy)	PD-(L)1 (N) CTLA-4 (I)
Ahmed et al. (30)	17	3	8.7	60	NSCLC	0.19	53	SRS (20 Gy), SRT (25 Gy)	PD-(L)1 (N, D)
Chen et al. (41)	260	2	9.2	NR	MBM (9%) NSCLC(79%) RCC (12%)	NR	61	SRS, SRT (20 Gy)	PD-(L)1 (P, N) CTLA-4 (I)
Anderson et al. (42)	36	1.5	9.2	67	MBM	NR	NR	SRS (21 Gy)	PD-(L)1 (P)
Fang et al. (43)	137	2	9.8	57	MBM	0.122	NR	SRS (20 Gy)	PD-(L)1 (P) CTLA-4 (I)
Kaidar-Person et al. (17)	58	2	12	59.5	MBM	NR	NR	SRS, FSRT (21 Gy)	PD-(L)1 (P, N) CTLA-4 (I)
Hubbeling et al. (27)	163	NR	16	61	NSCLC	NR	NR	SRS (18 Gy), PBI (30 Gy), WBRT (35 Gy)	PD-(L)1 (P, N, A)
Nardin et al. (45)	25	NR	8.4	58	MBM	0.43	72	SRS (20 Gy)	PD-(L)1 (P)
Du Four et al. (18)	142	NR	50	50	MBM	NR	NR	SRS (NR), WBRT (NR)	PD-(L)1 (P)
An et al. (46)	99	2	15.5	62.5	MBM	1.48	NR	SRS (20 Gy)	CTLA-4 (I)
Patel et al. (47)	54	1 (46%),2-3(40%), ≥3(13%)	NR	59.2	MBM	2.08	37	SRS (20 Gy)	CTLA-4 (I)
Silk et al. (48)	67	1(47%), 2(18%),≥3(49%)	NR	57.2	MBM	NR	44	SRS (14-24 Gy), WBRT (30-37 Gy)	CTLA-4 (I)
Tazi et al. (50)	10	3	NR	65.5	MBM	NR	NR	SRS (NR)	CTLA-4 (I)
Gaudy-Marqueste et al. (51)	68	NR	9.8	52.5	MBM	NR	NR	SRS (NR)	PD-(L)1 (NR) CTLA-4 (NR)
Kiess et al. (53)	46	2	22	57	MBM	NR	NR	SRS (21 Gy)	CTLA-4 (I)
Knisely et al. (54)	77	2	12.7	61	MBM	NR	58	SRS (NR)	CTLA-4 (I)
Cohen-Inbar et al. (55)	46	5	7.9	63	MBM	NR	80.4	SRS (20 Gy)	CTLA-4 (I)
Mathew et al. (56)	58	3	6	62	MBM	1.24	NR	SRS (20 Gy)	CTLA-4 (I)
Skrepnik et al. (26)	25	NR	22.7	68.5	MBM	NR	NR	SRS (21 Gy)	CTLA-4 (I)
Choong et al. (57)	65	NR	8.6	64.3	MBM	NR	NR	SRS (NR)	PD-(L)1 (NR) CTLA-4 (NR)
Williams et al. (58)	16	2	7.6	60	MBM	NR	50	SRS (24 Gy), WBRT (30 Gy)	CTLA-4 (I)
Yusuf et al. (59)	51	NR	7	63.6	MBM	0.18	NR	SRS (18 Gy)	PD-(L)1 (NR) CTLA-4 (NR)
Acharya et al. (60)	72	4	8.9	61	MBM	0.33	61	SRS (20 Gy)	PD-(L)1 (NR) CTLA-4 (NR)
Diao et al. (20)	91	NR	7.4	62	MBM	NR	NR	SRS (20 Gy)	CTLA-4 (I)
Martin et al. (39)	480	NR	23.6	61.8	-MBM (20%) NSCLC(70%) RCC(10%)	NR	NR	SRS, SRT (NR)	PD-(L)1 (P, N) CTLA-4 (I)
Kotecha et al. (61)	100	1	NR	61	MBM (17%) NSCLC(66%) RCC (12%) Other (5%)	NR	45	SRS (NR)	PD-(L)1 (NR)
Robin et al. (38)	38	1-3 (63%);≥4 (37%)	31.6	NR	MBM	NR	82	SRS (NR)	PD-(L)1 (NR) CTLA-4 (NR)
Gabani et al. (37)	1104	NR	6.42	62	MBM	NR	NR	SRS (20 Gy), WBRT (30 Gy)	PD-(L)1 (NR) CTLA-4 (NR)
Olson et al. (36)	24	NR	9	NR	MBM (54%) NSCLC(46%)	NR	NR	SRS (20 Gy)	PD-(L)1 (N, P)
Burke et al. (35)	26	NR	NR	NR	MBM	1.48	NR	SRS (21 Gy)	PD-(L)1 (N, P) CTLA-4 (I)
Johnson et al. (34)	37	2	15	NR	MBM (62%) NSCLC(27%) Other (11%)	1.1	NR	SRS (21 Gy)	PD-(L)1 (N) CTLA-4 (I)
Goel et al. (33)	51	NR	NR	NR	MBM	NR	NR	SRS (NR)	PD-(L)1 (NR)
Mortier (29)	57	NR	NR	NR	MBM	NR	NR	SRS (NR)	CTLA-4 (I)
Khoja et al. (31)	34	NR	7.4	NR	MBM	NR	NR	SRS (21 Gy), WBRT (NR)	CTLA-4 (I)
Silva et al. (32)	104	NR	24.3	56	MBM	NR	NR	SRS (20 Gy), WBRT (30 Gy)	PD-(L)1 (P, N)
Rahman et al. (28)	49	NR	NR	66	MBM	NR	84	(NR)	PD-(L)1 (NR)
Schapira et al. (52)	29	2	14.3	63	MBM	0.2	64.9	SRS (18 Gy)	PD-(L)1 (N, A, P)
Parakh et al. (48)	66	3	7	62	MBM	0.235	66	SRS (NR), WBRT (NR)	PD-(L)1 (N, P)
Stokes et al. (44)	429	NR	35.8	NR	MBM	NR	NR	SRS (NR), WBRT (NR)	PD-(L)1 (NR) CTLA-4 (NR)

*N (P), number of patients; N (L), number of lesions; median N (tL), median number of treated lesions; FU, follow-up; GTV, gross tumor volume; KPS, Karnofsky performance status; ECOG PS– Eastern Cooperative Oncology Group performance status; NR, not reported; MBM, melanoma brain metastases; NSCLC, non-small cell lung cancer; RCC, renal cell carcinoma; SRS, stereotactic radiosurgery; SRT, stereotactic radiotherapy; WBRT, whole-brain radiotherapy; PBI, partial brain irradiation; CTLA-4 - cytotoxic T-lymphocyte–associated antigen 4; PD-(L)1, Programmed death-(ligand)1; A, atezolizumab; D, durvalumab; I, ipilimumab; N, nivolumab; P, pembrolizumab; if patient data were reported for each group separately; a weighted average for the entire study was calculated*.

### Safety and Therapeutic Benefit of RT and ICI Combinations: Odds Ratio Analysis

To assess the overall benefit of RT and ICI combinations vs. RT alone, we first performed a meta-analysis of OR estimates. The 1-year OS OR, as derived from the available 13 studies with 2,450 patients enrolled, was significantly higher for the combination vs. RT alone (OR = 2.62 [95% CI: 1.92-3.58]; z = 6.08, *P* < 0.001, Supplementary Figure 4A). The funnel plot revealed an asymmetry (Supplementary Figure 5A; Egger test *P* = 0.0015), indicating potential publication bias regarding this particular outcome measure; significant heterogeneity was observed (I^2^ = 53.63%, Q = 32.87, *P* = 0.012). A meta-analysis of the 1-year LC OR, as derived from the available 4 studies with 463 patients enrolled, indicated no significant 1-year LC benefit for the combination (OR = 1.16 [95% I: 0.66-2.02]; z =0.52, *P* = 0.11, Supplementary Figure 4B). For this outcome, no asymmetry was detected on the funnel plot (Supplementary Figure 5B; Egger test *P* = 0.3) and no significant heterogeneity was observed (I^2^ = 38.58%, Q = 9.08, *P* = 0.11).

The impact of treatment on our selected safety outcome of RNR risk was evaluated based on information available from 10 studies with 1,196 patients enrolled; the meta-analysis did not allow us to exclude the possibility of an increase in RNR under RT and ICI combination therapy (OR = 1.75 [95% CI 1.02-2.99]; z = 2.04; *P* = 0.041, Supplementary Figure 4C). For this safety outcome, no publication bias was detected on the funnel plot (Supplementary Figure 5C; Egger test *P* = 0.46); no significant heterogeneity was observed (I^2^ = 23.28%, Q = 16.35, *P* = 0.23). It should be also noted that there was a higher number of patients treated with RT alone vs. RT and ICI combination, for all considered outcomes: 1-year OS (1,748 vs. 702 patients, respectively), 1-year LC (288 vs. 175 patients), and RNR (763 vs. 433 patients).

### Safety and Therapeutic Benefit of RT and ICI Combinations: Incidence Rate Analysis

As a next step, we performed a meta-analysis of the outcomes incidence rates. This allowed us to significantly increase the number of studies amenable to the analysis via inclusion of single-arm reports, which resulted in an increase in the relative proportion of patients treated with RT and ICI combination vs. RT alone. Also, in the study of such incidence rates, we experienced no publication bias: no funnel plot asymmetry was detected for outcomes rates (Egger test P values were 0.61, 0.88 and 0.058 for, respectively, 1-year OS, 1-year LC and RNR). Information on 1-year OS, 1-year LC and RNR was available from, respectively, 26, 6, and 23 studies (with, respectively, 3,101, 554, and 1,892 patients enrolled). Numbers of patients treated with RT alone vs. RT and ICI for 1-year OS, 1-year LC and RNR were, respectively, 1,748 vs. 1,353, 288 vs. 266 and 763 vs. 1,129.

In terms of overall survival, the combination was shown to be associated with a ~2-fold increase in 1-year OS; the corresponding incidence rates for the RT alone vs. RT and ICI groups were, respectively, 32% ([95% CI: 25-39%]; z = 15.67) vs. 59% ([95% CI: 54-63%]; z = 37.86) (covariate *P* < 0.0001). In contrast, local control was not affected by ICI inclusion into the RT treatment: 1-year LC incidence rates for the RT alone vs. RT and ICI groups were, respectively, 68% ([95% CI: 40-90%]; z = 6.77) vs. 72% ([95% CI: 63-80%]; z = 21.44) (covariate *P* = 0.72). Significant heterogeneity was observed in the analysis of both 1-year OS (I^2^ = 76.46%, Q = 216.65, heterogeneity *P* < 0.0001) and 1-year LC (I^2^ = 81.36%, Q = 64.39, heterogeneity *P* < 0.0001).

A meta-analysis on safety demonstrated no significant increase in RNR under combination therapy vs. RT alone. The estimated RNR values for RT alone vs. RT and ICI therapies were, respectively, 6% ([95% CI: 2-13%]; z = 4.26) vs. 9% ([95% CI: 5-14%]; z = 8.09) (covariate *P* = 0.37). Significant heterogeneity was observed for RNR (I^2^ = 83.24%, Q = 238.60, heterogeneity *P* < 0.0001).

### Evaluation of Factors Affecting Survival and Safety Outcomes in RT and ICI Combination Therapies

To further evaluate sources of the observed heterogeneity and identify factors affecting incidence rates of the considered outcomes, we used a mixed-effects meta-regression modeling approach. A list of the models we tested is given in Supplementary Table 4.

**Figure 2:**
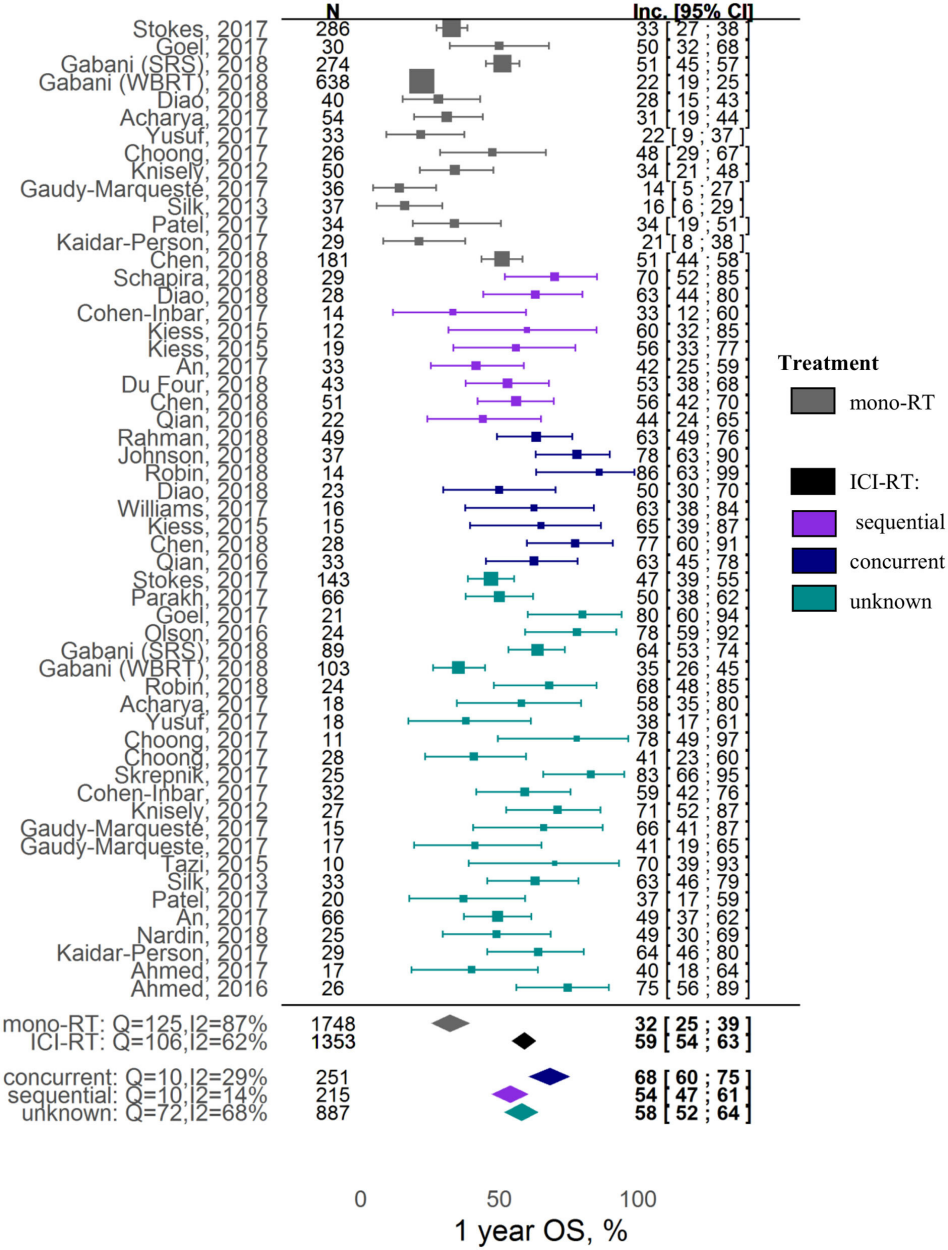
Forest plot: 1-year overall survival.

The key factor affecting the 1-year OS outcome most significantly was the sequencing of the RT and ICI combination: estimated incidence rates were 32% [95% CI: 25-39%], 54% [95% CI: 47-61%], 68% [95% CI: 60-75%], and 58% [95% CI: 52-64%] for, respectively, the RT alone, sequential RT+ICI, concurrent RT+ICI, and mixed (pooled or unknown sequencing) RT+ICI groups (Figure 2). Higher heterogeneity was observed in the RT alone group and the combination group with mixed scheduling regimens (respectively, I^2^ = 87%, Q =125, heterogeneity *P* < 0.0001; and I^2^ = 68%, Q = 72, heterogeneity *P* < 0.0001), compared to the combination groups with defined sequencing regimens (I^2^ = 29%, Q = 10, heterogeneity *P* = 0.18 for concurrent regimen; I^2^ = 10%, Q = 14, heterogeneity *P* = 0.27 for sequential regimen). No publication bias was detected upon examination of funnel plots (Egger test *P* = 0.61, Supplementary Figure 6). It should also be noted that the 1-year OS was higher for studies using only the SRS type of RT, as compared to studies using different types of RT which were pooled in the analysis (z = 1.86, covariate *P* = 0.0628). Interestingly, the significance of this covariate (SRS type of RT) was not confirmed during the sensitivity analysis; for example, exclusion of the Gabani et al. study from the analysis led to loss of statistical significance (*P* = 0.411), indicating a potential bias caused by the small sample size and high heterogeneity. Tumor histology and ICI type did not affect the 1-year OS outcome (Supplementary Table 4).

**Figure 3:**
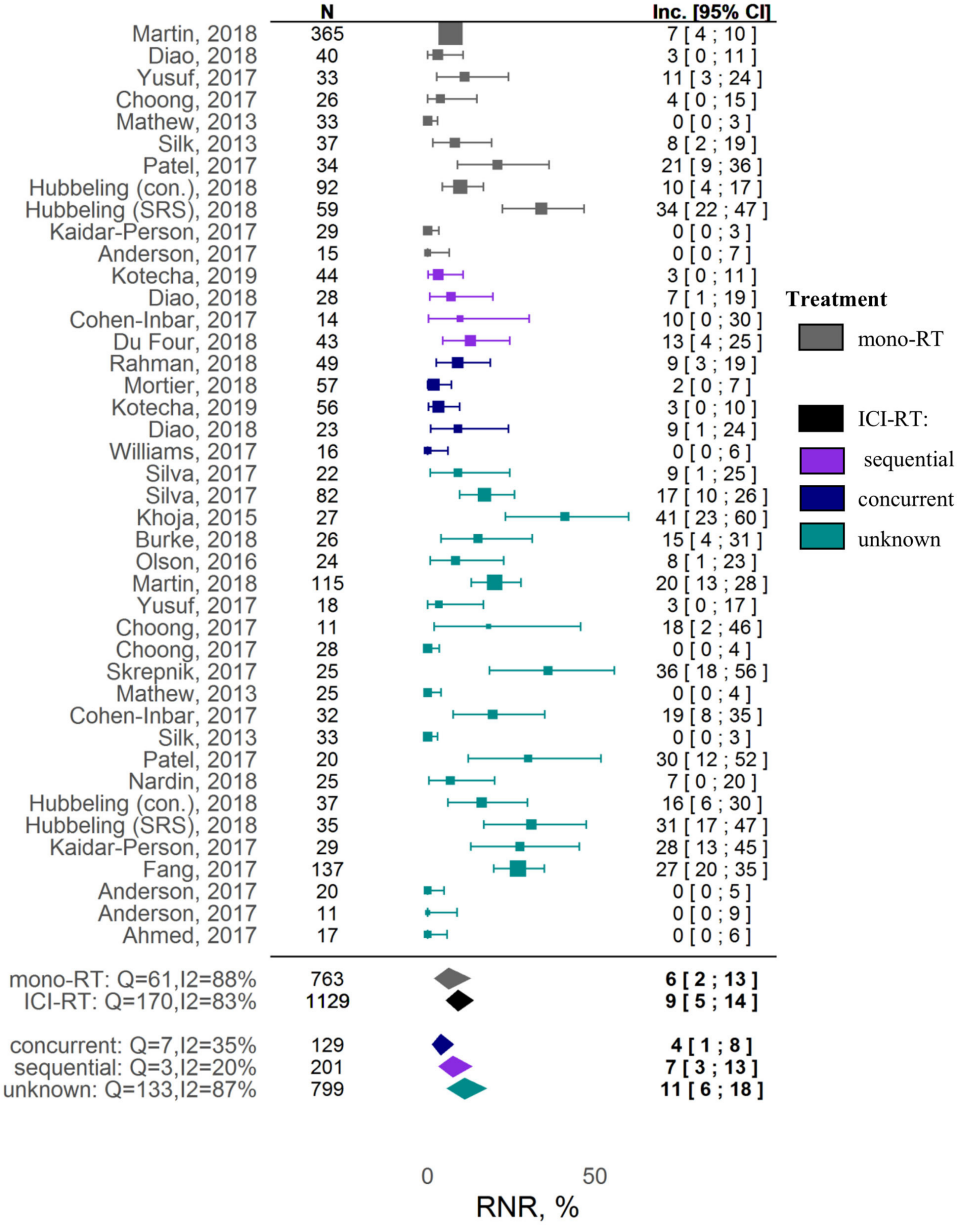
Forest plot: Radionecrosis rate (RNR).

No associations between different factors and other tested outcomes measures such as local control and RNR were found (Supplementary Table 4). A Forest plot for the 1-year LC outcome is available in Supplementary Figure 7; Forest plots for RNR are shown in Figure 3 and Supplementary Figure 9. The funnel plots (Supplementary Figure 6) indicate a potential publication bias for these outcomes (Egger test *P* values were 0.89 and 0.061 for, respectively, 1-year LC and RNR).

## Discussion

Our meta-analysis evaluated data from a total of 40 trials representing 4,359 patients with brain metastases originating mainly from melanoma and NSCLC. The analysis demonstrates that combined RT and ICI therapies are associated with a significant gain in overall survival, as compared to RT alone therapies, with a nearly two-fold increase in the milestone 1-year overall survival (32% [95% CI: 25-39%] vs. 59% [95% CI: 54-63%]), in line with results from other meta-analyses (63, 64). One limitation of the present analysis, when comparing OS in RT alone vs. ICI-RT, is that the latter form of treatment may result in improved overall systemic disease control due to a later enrollment into studies and access to novel medical technologies and treatment modalities.

Whereas, the current work is focused on the comparison of ICI-RT vs. RT monotherapies, efficacy and outcomes of ICI only therapies should also be considered. Results from numerous ICI only trials have been published (8), yet several factors need to be considered when interpreting these results, including small sample size, inclusion of RT-pretreated patients, and protocols allowing concurrent radiotherapy. In the first study (NCT00623766) which evaluated ICI activity in 72 melanoma patients (61% RT-pretreated patients) with symptomatic (cohort A, *N* = 51) and asymptomatic (cohort B, *N* = 21) metastases, 1-year OS was, respectively, 31 and 20% (65). An Anti-PD-1 Brain Collaboration (ABC) study (NCT02374242) which investigated efficacy of nivolumab alone or in combination with ipilimumab in a total of 76 RT-naïve melanoma brain metastasis (MBM) patients, 1-year OS was similar (60%) across the two cohorts (66); such an outcome is comparable to the outcome derived in our meta-analysis for ICI-RT combination treatments. In a phase II study (NCT02320058), a 1-year OS of 82.8% was observed, in 94 patients with MBM (9% RT-pretreated) and receiving a nivolumab and ipilimumab combination treatment (9). An Expanded Access Program (EAP) trial, which evaluated nivolumab efficacy in NSCLC, a 1-year OS of 43% was found, in patients with brain metastases (*N* = 409, 69% RT-treated) (67). A thorough analysis of individual patient-level data from these studies would provide more accurate quantitative estimates of a ICI-RT combination benefit. In contrast to a recent meta-analysis by Petrelli et al. (64), we used a meta-regression modeling approach to investigate the impact of different factors, including ICI and RT scheduling, ICI type and RT regimen, on the observed outcomes. This enabled us to extract incremental quantitative knowledge and to control for confounding factors. It should be stated that the lack of consistency, across retrospective studies, in the description of some of the patient baseline characteristics—including performance status, number of lesions and treatment history—limited our ability in testing the influence of these various factors on outcomes measures; however, it also provided further rationale for using a mixed-effects modeling methodology, in order to quantify heterogeneity in outcomes, which would arise from inter-study differences in patients enrolled.

Our analysis revealed that the scheduling of RT and ICI therapies, in the combination setting, plays a critical role, also in line with the mechanistic hypothesis we initially formulated based on preclinical data and quantitative mathematical modeling (3, 12, 13). Thus, concurrent treatment regimens were shown to be associated with higher milestone 1-year OS vs. sequenced treatment regimens: 69% [95% CI: 60-78%] vs. 52% [95% CI: 45-58%], respectively. This meta-analysis result is in full agreement with specific retrospective studies (12, 16, 19, 38, 40, 68). Interestingly, studies with a missing definition of the combination treatment regimen showed a nearly identical milestone 1-year OS vs. the overall OS estimate obtained without accounting for the regimen effect, respectively, 59% [95% CI: 52-66%] vs. 59% [95% CI: 54-64%], thereby providing additional validation of the results presented in this meta-analysis. One limitation in these results derived from a meta-analysis arises from a potential patient selection bias; subjects from sequential cohorts might have experienced a treatment change driven by disease progression and, therefore, may face a worse prognosis compared to patients under concurrent treatment. It should be also stated that a specific cut-off of 4 weeks was selected to differentiate between concurrent and sequential regimens, based on approximate half-lives of ICI drugs and in accordance with cut-off values selected from individual retrospective studies (20, 21). More precise estimates of the schedule timing vs. efficacy (outcomes) relationship may be obtained with access to individual patient-level continuous data, rather than operating with categorical data reported in the published literature.

Through this quantitative meta-analysis, we also determined that the type of ICI therapy (anti PD-(L)1, anti CTLA-4) did not significantly affect the milestone 1-year OS outcome, in line with other retrospective studies (38, 57). Choong et al. actually observed a trend of an improved OS for anti CTLA-4 vs. anti PD-1 agents, although this difference was not statistically significant (median OS=7.5 [95% CI: 4.4-15.6] and 20.4 [95% CI: 8.8-NA] months, respectively; (57)) and might relate to the small sample size of the study. Interestingly, evidence of an anti PD-(L)1 agent vs. an anti CTLA-4 agent benefit, when combined with RT, has been reported for other outcomes measures; for example, Robin et al. showed that RT and anti PD-1 alone or in combination with anti CTLA-4 was associated with higher PFS (*P* = 0.043) (38); Anderson et al. showed that the response rate of irradiated lesions was higher in patients treated with RT and anti PD-1 vs. RT and anti CTLA-4 (70 vs. 22%) (42). These data, taken together, indicate that either anti PD-(L)1, or anti CTLA-4, or their doublet may provide additional therapeutic benefits when combined with RT, but further studies are needed to determine optimal combination options and treatment regimens (10, 11).

Additionally, in clinical practice, the choice of an RT regimen is often dictated by other disease characteristics and symptoms, such as the number of brain metastases; this may further complicate the interpretation of outcomes from individual studies (69). For example, a lower milestone 1-year OS in patients treated with WBRT and ICI vs. patients treated with SRT regimens and ICI was found, in retrospective studies by Silk et al. (48), Gerber et al. (70), and Gabani et al. (37). However, the authors emphasized that this observation can be driven by confounding factors. For example, WBRT is typically warranted for patients with multiple metastases and thus with a corresponding poor outcome (37, 48, 70). The present meta-analysis indicated higher 1-year OS for SRT vs. other regimens, in both RT alone (35% [95% CI: 27-44%] vs. 24% [95% CI: 16-34%], respectively) and ICI-RT groups (60% [95% CI: 55-66%] vs. 54% [95% CI: 45-62%], respectively) (Supplementary Figure 8). In contrast, no differences in treatment effect on OS was observed, in two Phase III randomized trials (NCCTG N107C/CEC·3 and JCOG0504) comparing SRS vs. WBRT in combination with surgery (71, 72). This indicates that group differences, as observed in this retrospective analysis, might be due to differences in patient characteristics.

While combinations of ICI agents with RT appears to provide increased therapeutic benefits vs. RT alone, the possible potentiation of radio- and/or immuno-mediated toxicities is a significant concern, since there is clinical evidence and a mechanistic basis for additive toxicity in combination settings. For example, higher rates of treatment-related adverse events (any grade) were observed in the CA184-043 trial, which tested an ipilimumab (anti CTLA-4) combination with bone-directed RT vs. RT alone: 75 vs. 45%, respectively (73). In contrast to the above findings, no differences in treatment-related adverse event rates were found in a previously published retrospective analysis evaluating safety and therapeutic benefit of nivolumab (anti PD-1) alone vs. in combination with HFRT in NSCLC (74).

Given the variety of safety outcomes, we decided to focus on RNR, as this adverse effect is RT-specific and is reported systematically across most studies. Even though the pathogenesis of radionecrosis is not fully characterized, activation of pro-inflammatory mechanisms is associated with vascular injury and abnormal angiogenesis; hence, there may be concerns about worsening of RT-mediated neurological symptoms, with an added ICI treatment (75). In our meta-analysis, however, combinations of ICI and RT vs. RT alone were not associated with a significant increase in RNR. It should be noted that RNR exhibited a high degree of heterogeneity among the studies included in the present meta-analysis, possibly reflecting challenges and patient-to-patient variability in the reporting of this adverse event. For example, in a study by Martin et al., the RNR was significantly higher in the combination setting vs. the RT alone group (20% [95% CI: 13-28%] vs. 7.0% [95% CI: 4-10%]) (39), whereas no radionecrosis episodes were observed in combination cohorts in multiple other studies (30, 42, 48). Such a high variability in reported RNR may be due to different definitions used to determine radionecrosis, on the basis of MRI images, histopathological changes, and/or clinical symptoms (17, 30, 42, 43), and also to difficulties in distinguishing progression from pseudo-progression when determining radionecrosis (72). Another potential confounding factor in analyses invoking RNR may relate to differences in follow-up times across studies, given radionecrosis may manifest itself around 6 to 30 months following RT, whereas the average of median follow-up times across studies was 13.3 months (18, 42). Additionally, the type of RT administered also affects neurotoxicity; for example, WBRT has been shown to be associated with faster cognitive decline (71); Hubeling et al. reported higher RNR in SRS vs. PBI- and WBRT-treated patients in both RT alone and combination groups (27). Our analysis did not allow us to identify differences between stereotactic and pooled RT regimens in terms of RNR in both RT alone and ICI-RT groups (Supplementary Figure 10). However, this could be a result of the aforementioned heterogeneity and lack of detailed information on RT regimens available from individual studies.

In summary, the present meta-analysis demonstrated outcomes benefits and comparable RNR safety profiles, when combining RT and ICI treatments vs. RT monotherapy, in line with previous findings from individual clinical trials and other, less comprehensive meta-analyses. However, given the aforementioned limitations, in particular the retrospective nature of the observations collected, these results may be viewed as a hypothesis which would require confirmation in prospective randomized trials, e.g., comparing concurrent and sequential RT and ICI treatment vs. the corresponding ICI standard of care (e.g., CTLA-4 Ab, PD-1/PD-L1 Ab or their combination), in patients within an indication and with same disease status. Meanwhile, an analysis based on the availability of individual outcomes data in RT-pretreated patients from past trials [e.g., ABC study/NCT02374242; EAP study (67)] would allow for a further quantitative understanding of ICI-RT outcomes in the treatment of metastatic brain disease.

Lastly, it should be stated that the present work is focused on particular aspects of IO-RT treatment optimization, however, given the complex landscape of drug development in the immuno-oncology arena (76), a few other directions for further research exist. Recent clinical studies aim at evaluating new ICI combination strategies, including ICI drug combinations with chemotherapies, chemo-radiotherapies, or targeted treatments such as bevacizumab (NCT02681549, NCT03175432), for management of brain metastases. Also, the bulk of the current clinical evidence for ICI-RT combination efficacy is based on observations from melanoma and NSCLC metastatic disease, while other indications are currently under investigation, including metastatic breast cancer (NCT03483012, NCT03807765, NCT03449238) and leptomeningeal carcinomatosis [NCT02886585 (77)]. Future research should also aim at investigating the efficacy of novel immunotherapies entering the clinical space, identifying predictive biomarkers used for patient selection, and defining a strategy to mitigate the risk of radionecrosis and other treatment-related adverse events (8).

## Data Availability Statement

The datasets generated for this study are available on request to the corresponding author.

## Author Contributions

All authors made a substantial contribution to the research. VV, KP, and GH: conceptualization. VV and KP: methodology. VV and KP: formal analysis. VV, KP, MS, and SL: investigation. VV and KP: writing—original draft. All authors: writing—review & editing. VV: visualization. MS, SL, and GH: supervision.

## Conflict of Interest

VV and KP are employed by M&S Decisions LLC. GH is employed by Obsidian Therapeutics. The remaining authors declare that the research was conducted in the absence of any commercial or financial relationships that could be construed as a potential conflict of interest.
