# Supplementary material for: Quantification of Scheduling Impact on Safety and Efficacy Outcomes of Brain Metastasis Radio- and Immuno-Therapies: A Systematic Review and Meta-Analysis
Source: Front Oncol. 2020 Sep 2;10:1609. doi: 10.3389/fonc.2020.01609 (PMC7492564; doi:10.3389/fonc.2020.01609)
Supplement: Supplementary file 1 [file Table_1.DOCX]

Supplementary Material to the manuscript

*Quantification of scheduling impact on safety and efficacy outcomes of brain metastasis radio- and immuno-therapies: a systematic review and meta-analysis*

# Supplementary Tables

**Supplementary Table 1.** PRISMA checklist

| **Section/topic** | **#** | **Checklist item** | **Reported on page #** |
| --- | --- | --- | --- |
| **TITLE** | | |  |
| Title | 1 | Identify the report as a systematic review, meta-analysis, or both. | 1 |
| **ABSTRACT** | | |  |
| Structured summary | 2 | Provide a structured summary including, as applicable: background; objectives; data sources; study eligibility criteria, participants, and interventions; study appraisal and synthesis methods; results; limitations; conclusions and implications of key findings; systematic review registration number. | 1 |
| **INTRODUCTION** | | |  |
| Rationale | 3 | Describe the rationale for the review in the context of what is already known. | 2 |
| Objectives | 4 | Provide an explicit statement of questions being addressed with reference to participants, interventions, comparisons, outcomes, and study design (PICOS). | 2 |
| **METHODS** | | |  |
| Protocol and registration | 5 | Indicate if a review protocol exists, if and where it can be accessed (e.g., Web address), and, if available, provide registration information including registration number. | No protocol exists |
| Eligibility criteria | 6 | Specify study characteristics (e.g., PICOS, length of follow-up) and report characteristics (e.g., years considered, language, publication status) used as criteria for eligibility, giving rationale. | 3 |
| Information sources | 7 | Describe all information sources (e.g., databases with dates of coverage, contact with study authors to identify additional studies) in the search and date last searched. | 2 |
| Search | 8 | Present full electronic search strategy for at least one database, including any limits used, such that it could be repeated. | 3 |
| Study selection | 9 | State the process for selecting studies (i.e., screening, eligibility, included in systematic review, and, if applicable, included in the meta-analysis). | 4 |
| Data collection process | 10 | Describe method of data extraction from reports (e.g., piloted forms, independently, in duplicate) and any processes for obtaining and confirming data from investigators. | 2 |
| Data items | 11 | List and define all variables for which data were sought (e.g., PICOS, funding sources) and any assumptions and simplifications made. | 3 |
| Risk of bias in individual studies | 12 | Describe methods used for assessing risk of bias of individual studies (including specification of whether this was done at the study or outcome level), and how this information is to be used in any data synthesis. | 4 |
| Summary measures | 13 | State the principal summary measures (e.g., risk ratio, difference in means). | 3 |
| Synthesis of results | 14 | Describe the methods of handling data and combining results of studies, if done, including measures of consistency (e.g., I^2^) for each meta-analysis. | 4 |

**Supplementary Table 2.** MOOSE checklist

| **Item No** | **Recommendation** | **Reported on Page No** |
| --- | --- | --- |
| Reporting of background should include | | |
| 1 | Problem definition | 2 |
| 2 | Hypothesis statement | 2 |
| 3 | Description of study outcome(s) | 2 |
| 4 | Type of exposure or intervention used | 2 |
| 5 | Type of study designs used | 2 |
| 6 | Study population | 2 |
| Reporting of search strategy should include | | |
| 7 | Qualifications of searchers (e.g., librarians and investigators) | 2 |
| 8 | Search strategy, including time period included in the synthesis and key words | 2-3 |
| 9 | Effort to include all available studies, including contact with authors | 3 |
| 10 | Databases and registries searched | 2 |
| 11 | Search software used, name and version, including special features used (e.g., explosion) | NA |
| 12 | Use of manual searching (e.g., reference lists of obtained articles) | 3 |
| 13 | List of citations located and those excluded, including justification | 4 |
| 14 | Method of addressing articles published in languages other than English | 3 |
| 15 | Method of handling abstracts and unpublished studies | 3 |
| 16 | Description of any contact with authors | 3 |
| Reporting of methods should include | | |
| 17 | Description of relevance or appropriateness of studies assembled for assessing the hypothesis to be tested | 4 |
| 18 | Rationale for the selection and coding of data (e.g., sound clinical principles or convenience) | 3 |
| 19 | Documentation of how data were classified and coded (e.g., multiple raters, blinding and interrater reliability) | 3-4 |
| 20 | Assessment of confounding (e.g., comparability of cases and controls in studies where appropriate) | 4 |
| 21 | Assessment of study quality, including blinding of quality assessors, stratification or regression on possible predictors of study results | 4 |
| 22 | Assessment of heterogeneity | 4 |
| 23 | Description of statistical methods (e.g., complete description of fixed- or random-effects models, justification of whether the chosen models account for predictors of study results, dose-response models, or cumulative meta-analysis) in sufficient detail to be replicated | 4 |
| 24 | Provision of appropriate tables and graphics | Figures 1-3, Table 1 |
| Reporting of results should include | | |
| 25 | Graphic summarizing individual study estimates and overall estimate | Figures 2, 3 |
| 26 | Table giving descriptive information for each study included | Table 1 |
| 27 | Results of sensitivity testing (e.g., subgroup analysis) | Figures 2, 3 |
| 28 | Indication of statistical uncertainty of findings | Figures 2, 3 |
| Reporting of discussion should include | | |
| 29 | Quantitative assessment of bias (e.g., publication bias) | Figures S5, S6 |
| 30 | Justification for exclusion (e.g., exclusion of non-English language citations) | 6-7 |
| 31 | Assessment of quality of included studies | 6-7 |
| Reporting of conclusions should include | | |
| 32 | Consideration of alternative explanations for observed results | 6-7 |
| 33 | Generalization of the conclusions (i.e., appropriate for the data presented and within the domain of the literature review) | 8 |
| 34 | Guidelines for future research | 8 |
| 35 | Disclosure of funding source | 9 |

**Supplementary Table 3.** Summary of studies by covariate values

| **1-year OS** | | |
| --- | --- | --- |
| **Stratification** | **N of Arms** | **Total N of arms** |
| By scheduling | Concurrent: 8  Sequential: 9  Unknown: 24  Mono-RT: 14 | 55 |
| By ICI class | PD-(L)1 Ab: 10  CTLA-4 Ab: 17  Mixed: 14  Mono-RT: 14 |  |
| By RT type | SRS: 45  PBI-WBRT-SRS: 10 |  |
| By histology | MBM: 48  NSCLC, pooled: 7 |  |
| **1-year LC** | | |
| **Stratification** | **N of Arms** | **Total N of arms** |
| By scheduling | Concurrent: 3  Sequential: 3  Unknown: 4  Mono-RT: 4 | 14 |
| By ICI class | PD-(L)1 Ab: 2  CTLA-4 Ab: 4  Mixed: 4  Mono-RT: 4 |  |
| By RT type | SRS: 14  PBI-WBRT-SRS: 0 |  |
| By histology | MBM: 9  NSCLC, pooled: 5 |  |
| **RNR** | | |
| **Stratification** | **N of Arms** | **Total N of arms** |
| By scheduling | Concurrent: 5  Sequential: 4  Unknown: 22  Mono-RT: 11 | 42 |
| By ICI class | PD-(L)1 Ab: 13  CTLA-4 Ab: 13  Mixed: 5  Mono-RT: 11 |  |
| By RT type | SRS: 33  PBI-WBRT-SRS: 9 |  |
| By histology | MBM: 32  NSCLC, pooled: 10 |  |

**Supplementary Table 4.** List of tested models

| **1-year OS** | | | | | | |
| --- | --- | --- | --- | --- | --- | --- |
| **Model formula** | **df** | **I^2^, %** | **Q** | **pval** | **AICc** | **pval, covariate** |
| - (base model) | - | 87.11 | 418.85 | < .0001 | -20.97 | intrcpt. <0 .0001 |
| ~Combo | 1 | 77.04 | 230.81 | < .0001 | -48.67 | intrcpt. < 0.0001; ICItr<0 .0001 |
| ~ SCH | 3 | 76.46 | 216.65 | < .0001 | -48.22 | intrcpt. < 0.0001; conc.<0.0001; seq=0.001; unknown<0 .0001 |
| ~ PD | 3 | 75.55 | 227.2 | < .0001 | -45.18 | intrcpt. < 0.0001; CTLA4 Ab <0.0001; PD-(L)1 Ab <0.0001; mixed <0.0001 |
| ~RT | 1 | 81.81 | 291.41 | < .0001 | -21.61 | intrcpt. < 0.0001; SR=0.06 |
| ~Histology | 1 | 85.73 | 371.42 | < .0001 | -23 | intrcpt. < 0.0001; MBM=0.0442 |
| ~SCH+RT | 4 | 65.13 | 143.37 | < .0001 | -53.22 | intrcpt. < 0.0001; conc.<0.0001; seq=0.0004; unknown<0 .0001; SR=0.01 |
| ~SCH+PD | 5 | 76.98 | 212.88 | < .0001 | -44.38 | intrcpt. < 0.0001; conc.<0.0001; seq=0.006; unknown<0 .0001; CTLA4 Ab=0.37; PD-(L)1 Ab =0.39 |
| ~SCH + Histology | 4 | 72.55 | 182.14 | < .0001 | -51.11 | intrcpt. < 0.0001; conc.<0.0001; seq=0.0021; unknown<0 .0001; MBM=0.04 |
| **1-year LC** | | | | | | |
| **Model formula** | **df** | **I^2^, %** | **Q** | pval | **AICc** | **pval, covariate** |
| - (base model) | - | 79.96 | 64.88 | < .0001 | -2.4 | intrcpt. < 0.0001 |
| ~Combo | 1 | 81.36 | 64.39 | < .0001 | 1.01 | intrcpt. < 0.0001; ICItr=0.72 |
| ~ SCH | 3 | 83.1 | 59.19 | < .0001 | 9.18 | intrcpt. < .0001, conc.=0.43; seq=0.69; unknown=0.80 |
| ~ PD | 3 | 81.02 | 52.68 | < .0001 | 7.1 | intrcpt. < .0001; CTLA4 Ab =0.58; PD-(L)1 Ab=0.13; mixed =0.25 |
| ~RT | no observations | | | | | |
| ~Histology | 2 | 74.17 | 46.46 | < .0001 | -1.15 | intrcpt. < 0.0001, MBM=0.11 |
| **RNR** | | | | | | |
| **Model formula** | **df** | **I^2^, %** | **Q** | pval | **AICc** | **pval, covariate** |
| - (base model) |  | 82.85 | 239.13 | < .0001 | -9.16 | intrcpt. <0.0001 |
| ~Combo | 1 | 82.68 | 230.95 | < .0001 | -7.65 | intrcpt. < .0001; ICItr=0.37 |
| ~ SCH | 3 | 81.32 | 203.39 | < .0001 | -5.05 | intrcpt. < 0.0001; conc.=0.27; seq=0.90; unknown=0.10 |
| ~ PD | 3 | 80.18 | 191.77 | < .0001 | -5.69 | intrcpt. < 0.0001; CTLA4 Ab =0.7215; PD-(L)1 Ab=0.62; mixed =0.06 |
| ~RT | 1 | 83.27 | 239.06 | < .0001 | -7.03 | intrcpt. <0.0001, SR=0.84 |
| ~Histology | 2 | 83.24 | 238.6 | < .0001 | -7.92 | intrcpt. < 0.0001, MBM=0.36 |
| **1-year OS: censoring impact** | | | | | | |
| **Model formula** | **df** | **I^2^, %** | **Q** | pval | **AICc** | **pval, covariate** |
| - (base model) | 42 | 87.23 | 32.98 | < .0001 | -14.28 | intrcpt. < 0.0001 |
| ~censoring | 1 | 85.68 | 28.63 | < .0001 | -14.93 | intrcpt. < .0001, cens.=0.11 |

Selected models are shown in grey shading; intrcpt. – intercept, covariates: ICI-RT – combined ICI-RT treatment (comparison to mono-RT); conc., seq. unknown – concurrent, sequential and unknown ICI-RT scheduling, respectively (comparison to mono-RT); PD-(L)1 Ab, CTLA-4 Ab – RT combination with PD-(L)1 Ab and CTLA-4 Ab, respectively(comparison to mono-RT); MBM – melanoma brain metastases (comparison to pooled or non-small cell lung cancer brain metastases); SR – stereotactic regimens (comparison to pooled RT); cens. – censoring.

# Supplementary figures


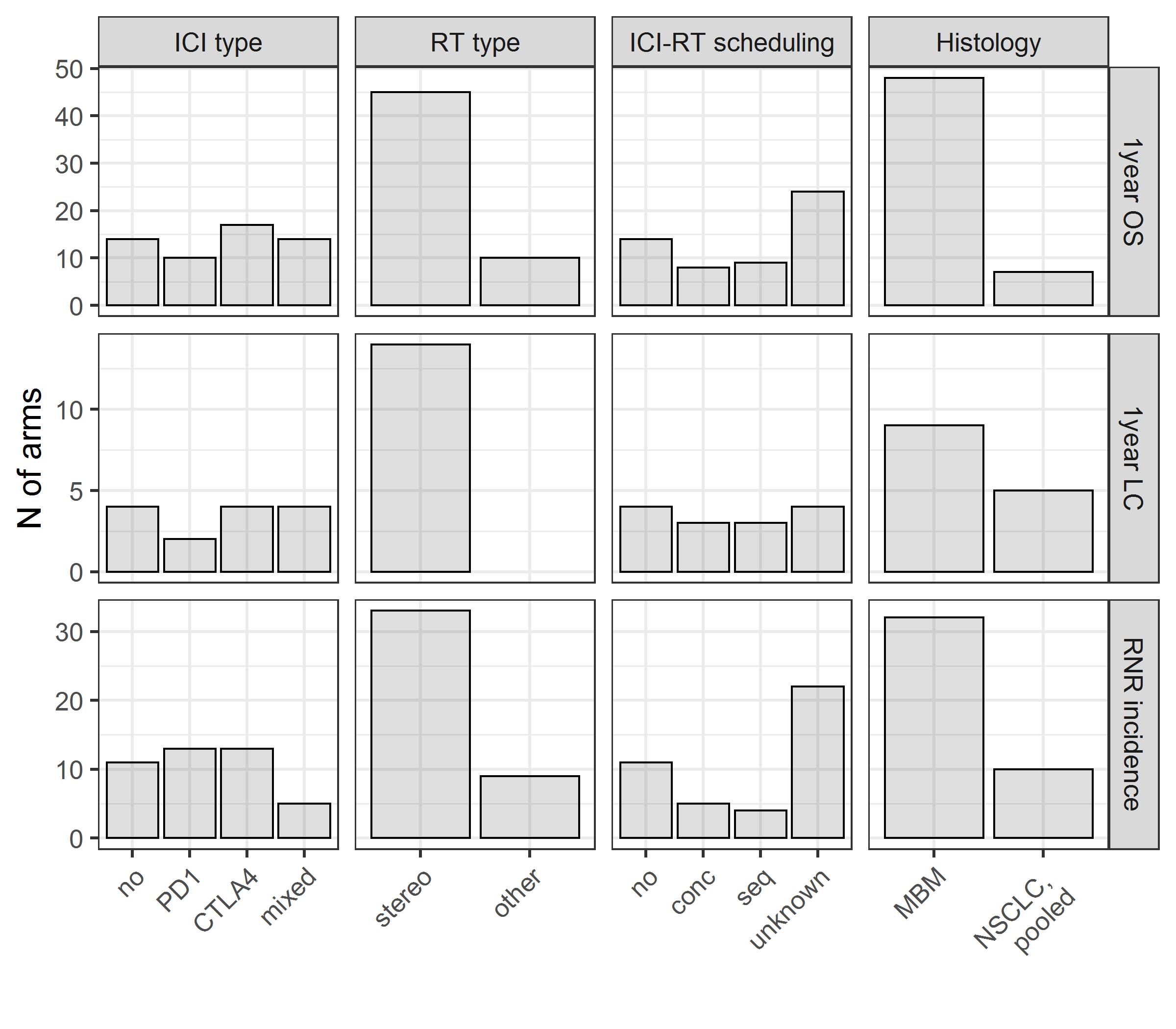


**Supplementary Figure 1.** Study arm stratification by covariate values


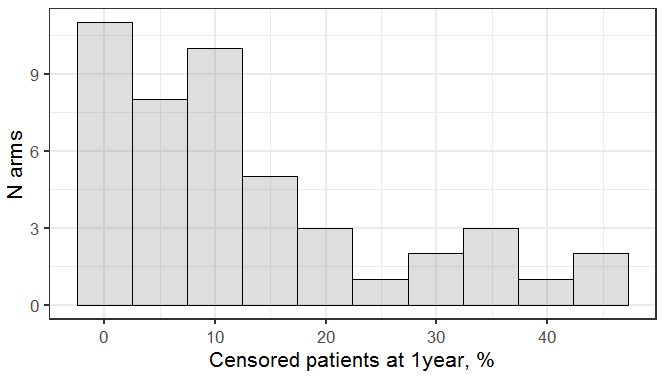


**Supplementary Figure 2.** Censoring intensity in the considered studies


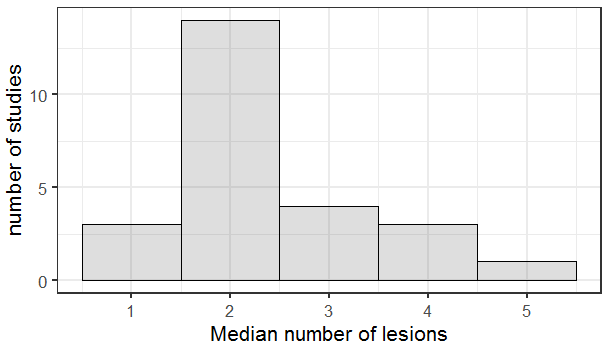


**Supplementary Figure 3.** Median number of lesions in the considered studies


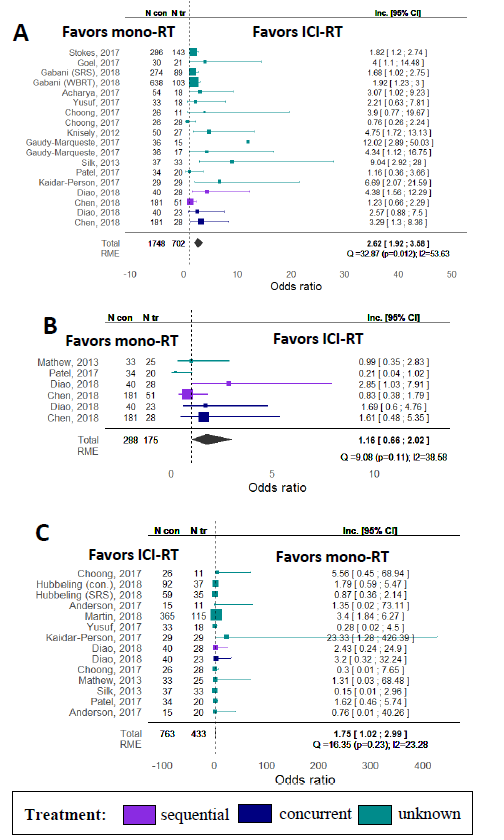


**Supplementary Figure 4.** OR forest plots for: **(A)** 1-year OS; **(B)** 1-year LC; **(C)** RNR


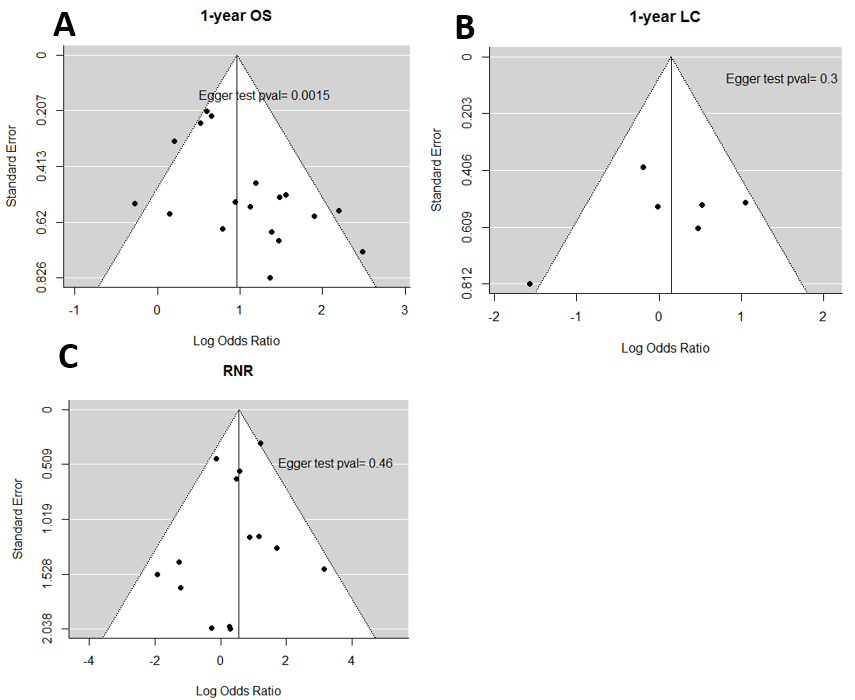


**Supplementary Figure 5.** Funnel plots for: **(A)** 1-year OS; **(B)** 1-year LC; **(C)** RNR


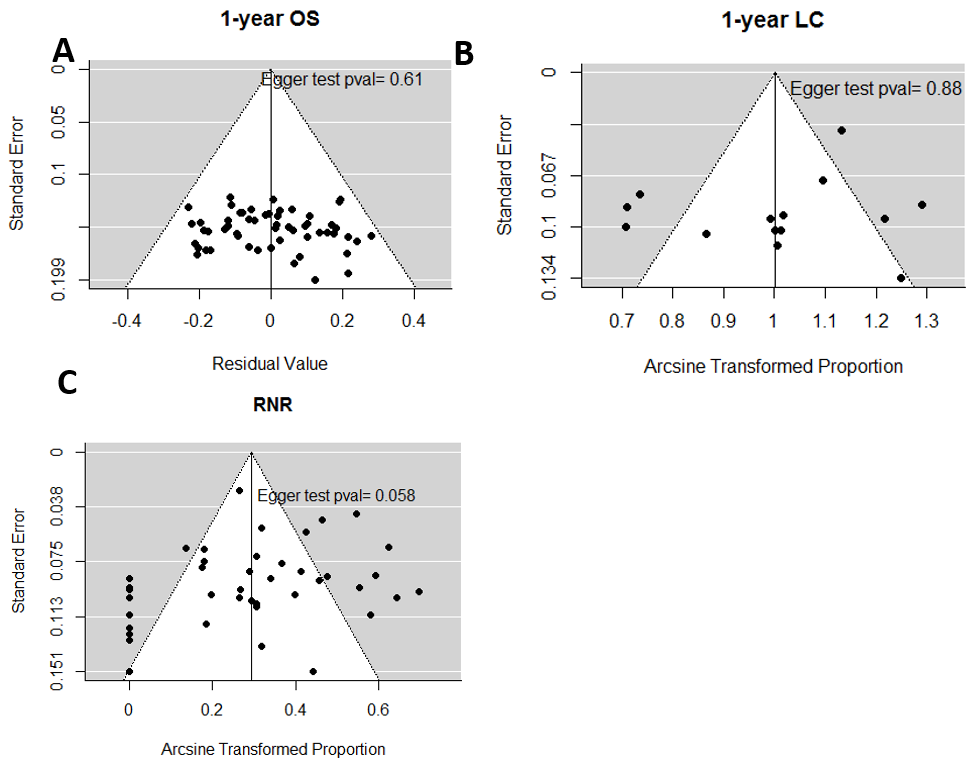


**Supplementary Figure 6.** Funnel plots for the final modes (Table S3): (**A)** 1-year OS (covariate on sequencing regimen); **(B)** 1-year LC (no covariates); **(C)** RNR (no covariates)


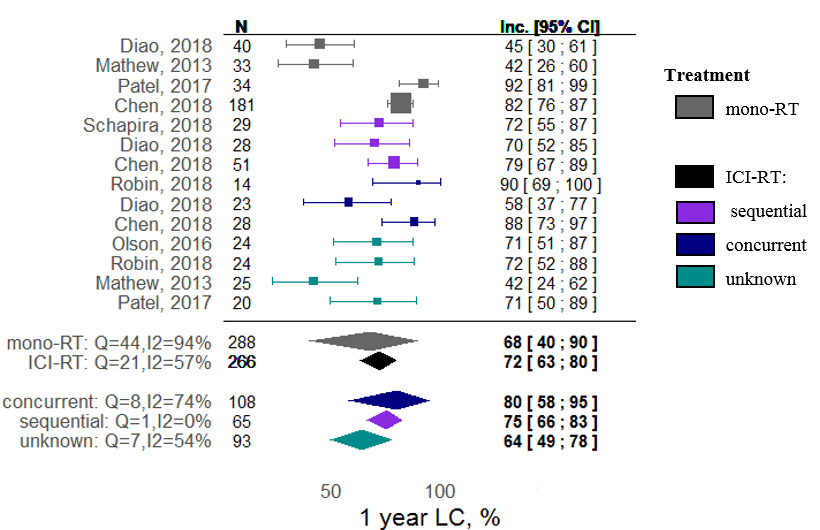


**Supplementary Figure 7.** Forest plot: 1-year LC


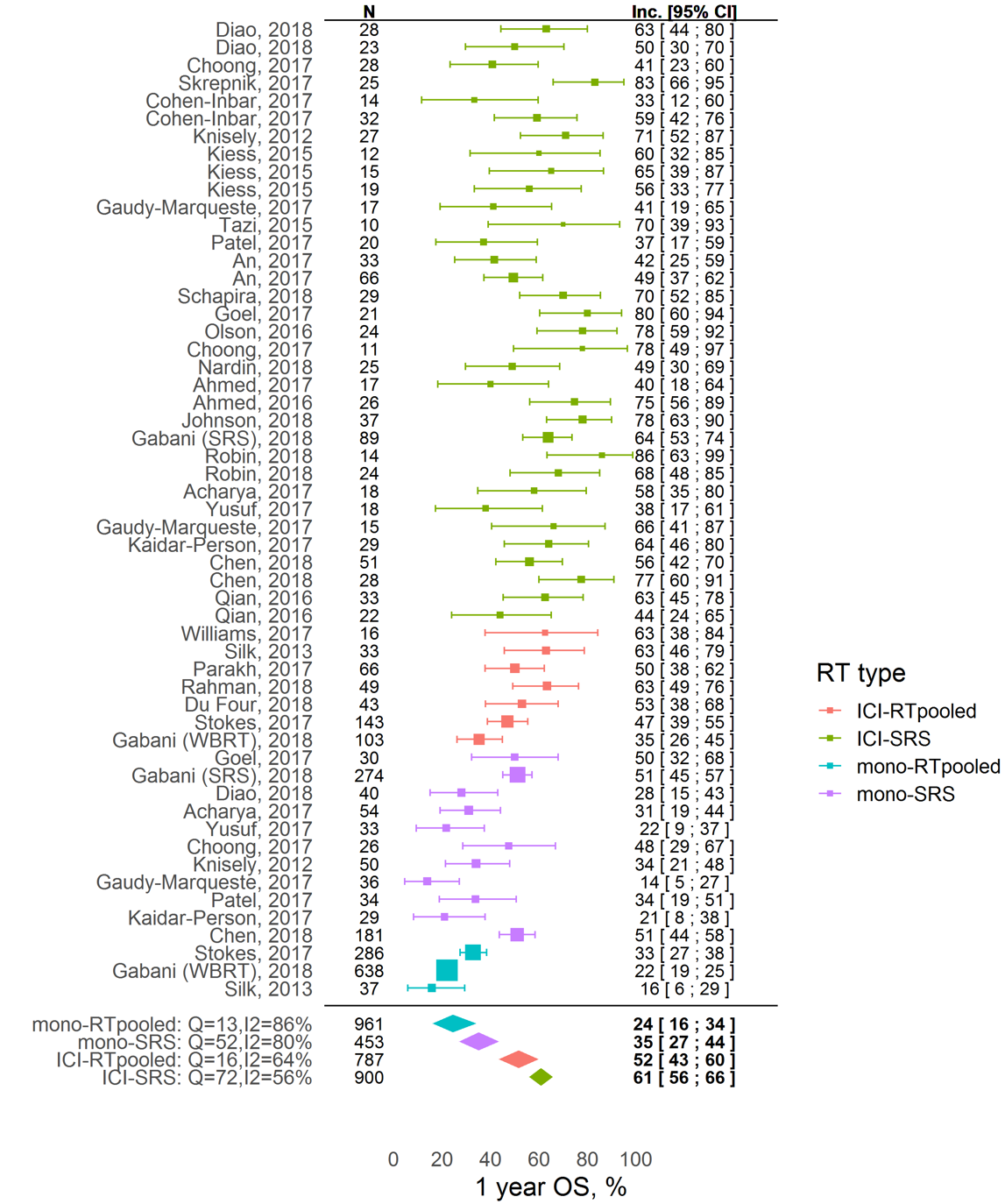


**Supplementary Figure 8.** Forest plot: 1-year OS, stratified by RT type and ICI administration


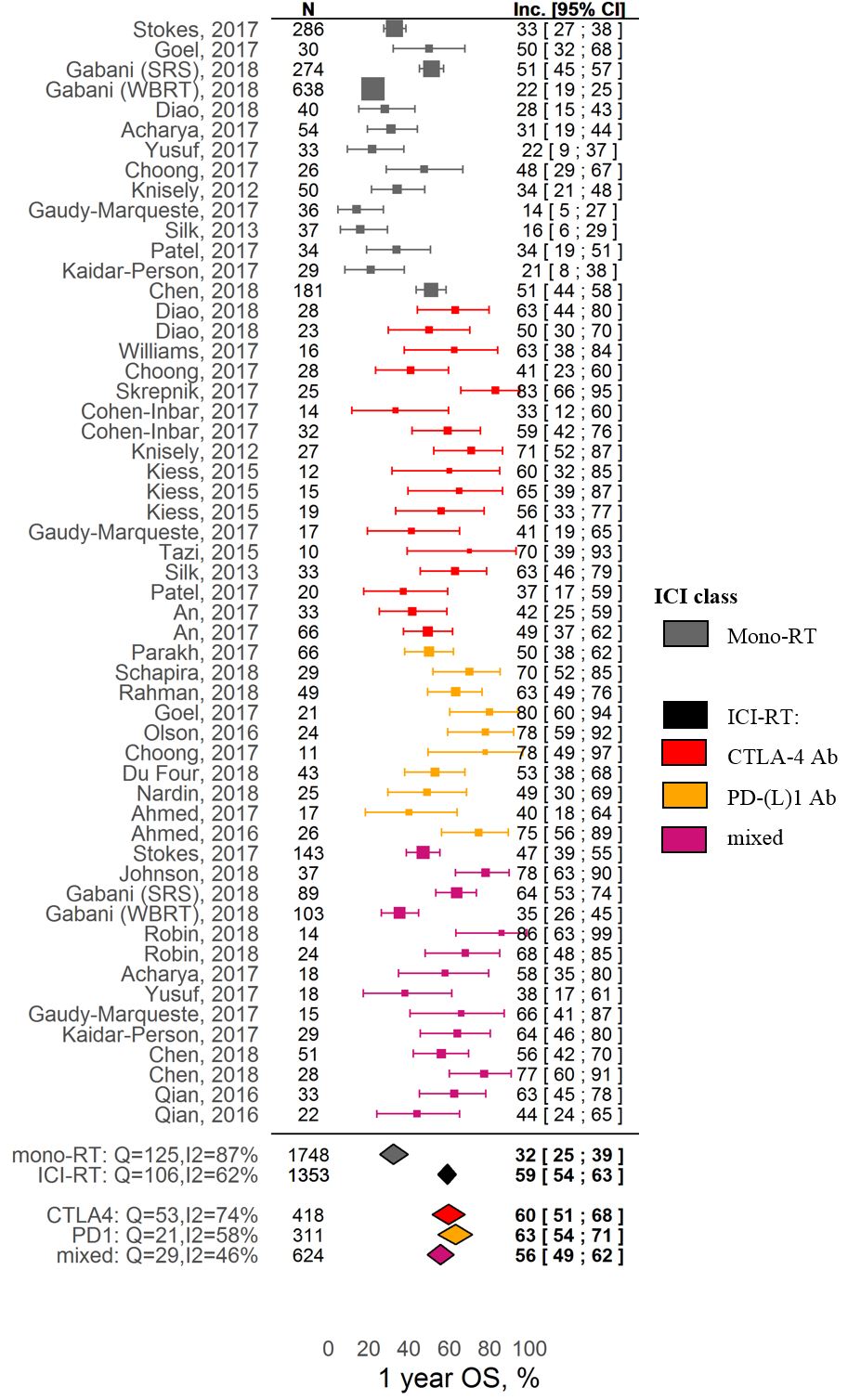


**Supplementary Figure 9.** Forest plot: 1-year OS, stratified by ICI administration and type


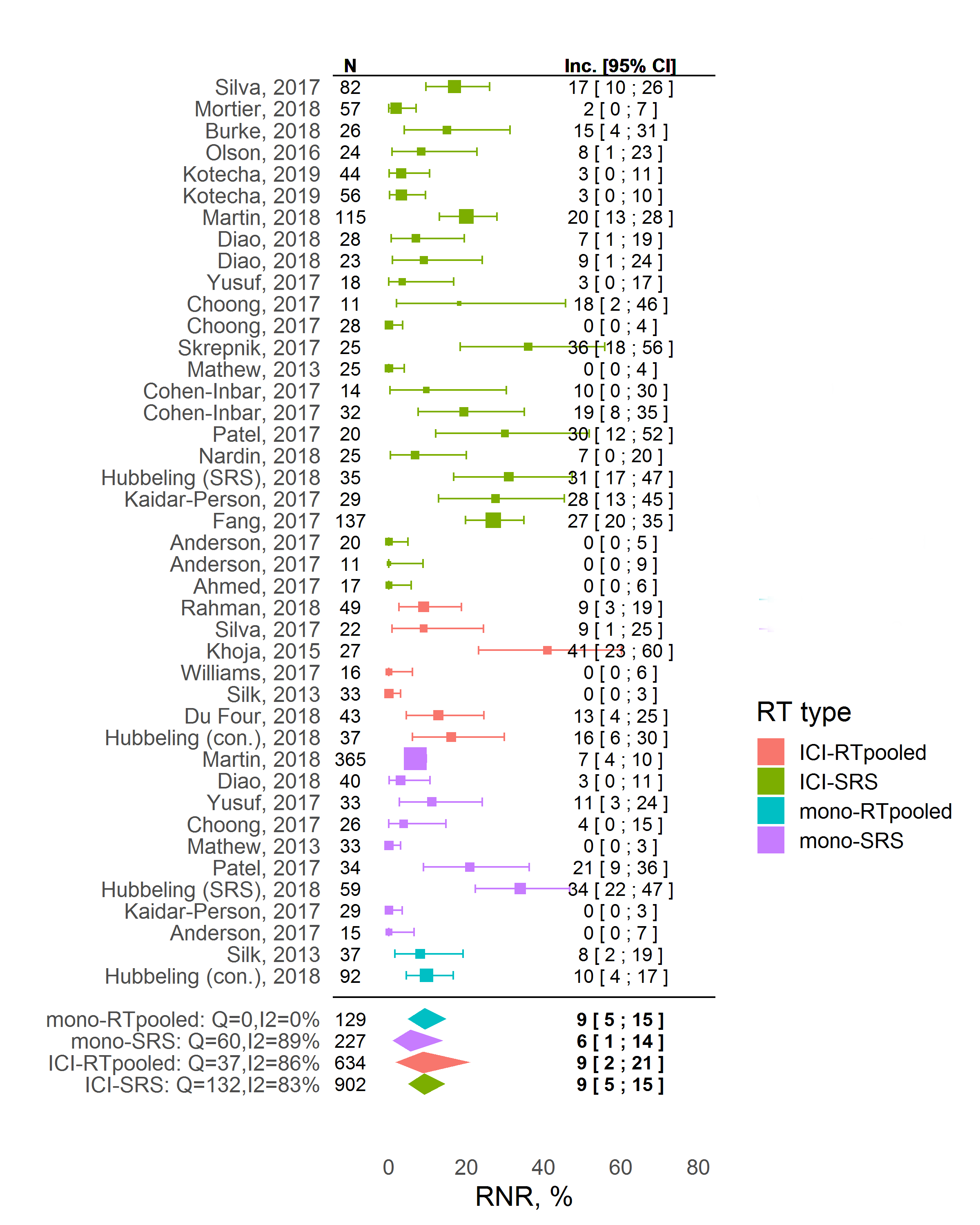


**Supplementary Figure 10.** Forest plot: RNR, stratified by RT type and ICI administration


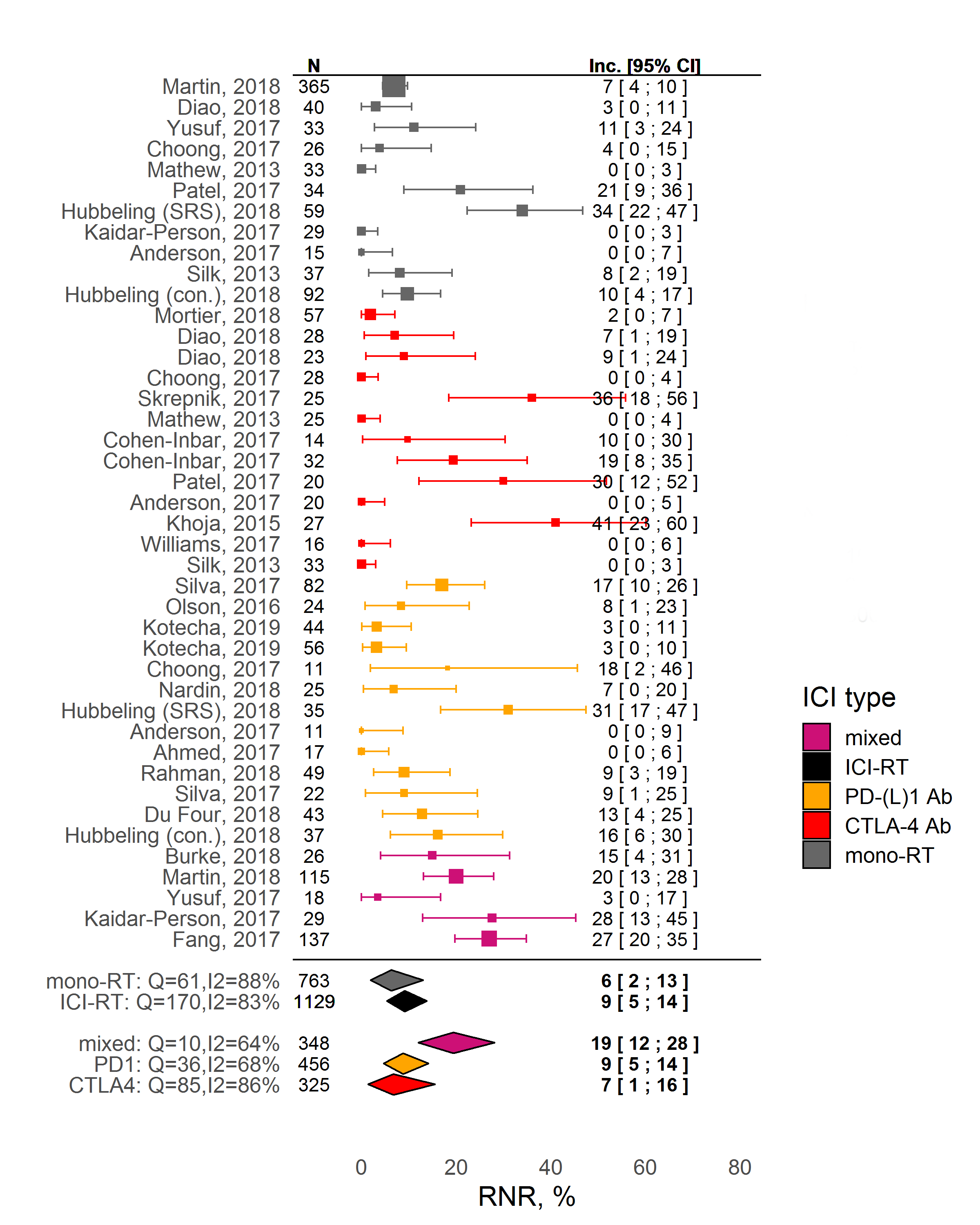


**Supplementary Figure 11.** Forest plot: RNR, stratified by ICI administration and type


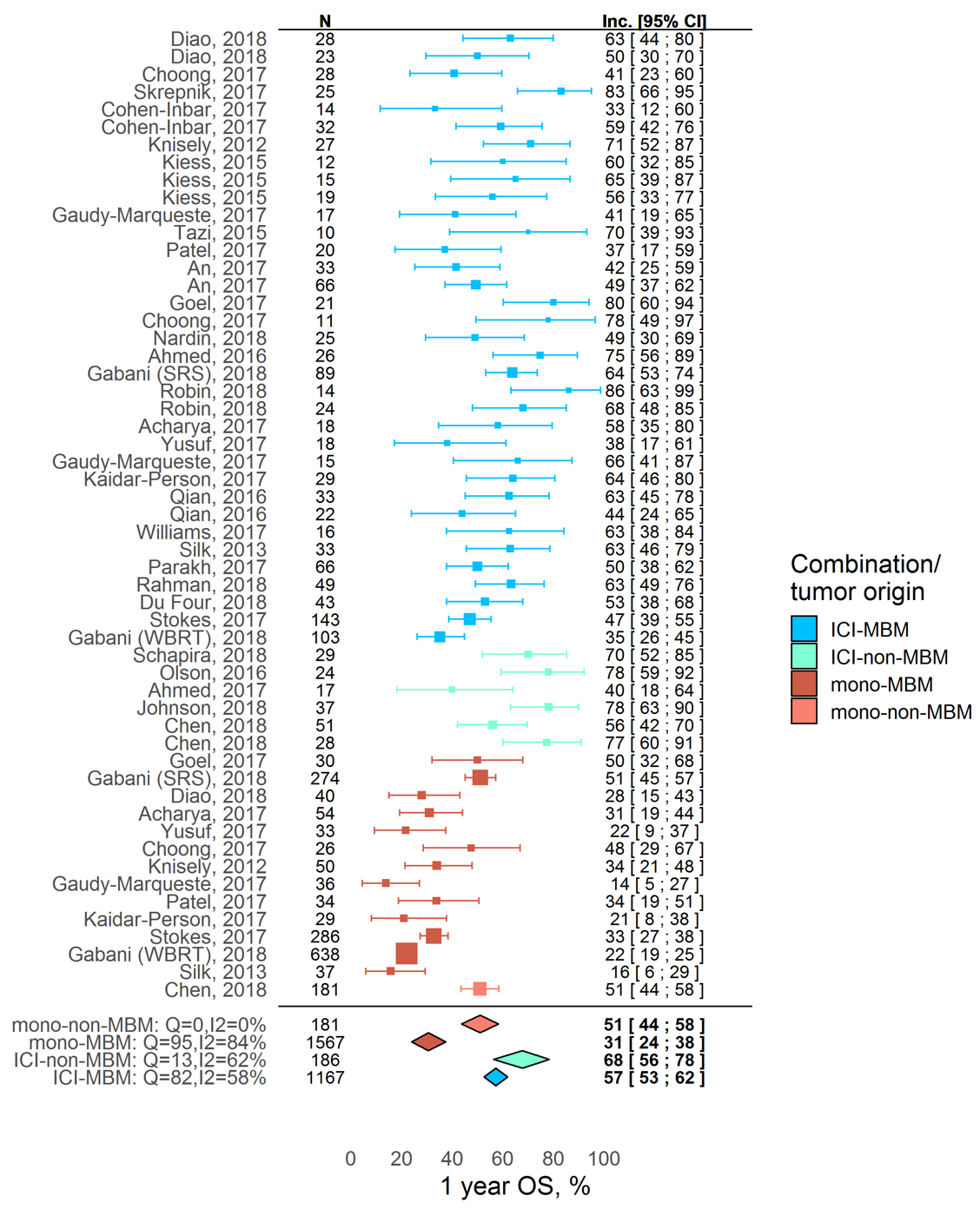


**Supplementary Figure 12.** Forest plot: 1-year OS, stratified by ICI administration and tumor origin
